# Netrins and Their Roles in Placental Angiogenesis

**DOI:** 10.1155/2014/901941

**Published:** 2014-07-17

**Authors:** Mbarka Dakouane-Giudicelli, Nadia Alfaidy, Philippe de Mazancourt

**Affiliations:** ^1^Université de Versailles Saint Quentin en Yvelines, Unité Pathologie Cellulaire et Génétique (UPCG) UPRES EA24-93, UFR des Sciences de la Santé, 2 avenue de la Source de la Bièvre, 78180 Montigny le Bretonneux, France; ^2^Unité Mixte INSERM-UJF-CEA-CNRS U1036, CEA, 17 rue des Martyrs, 38054 Grenoble, France

## Abstract

Netrins, a family of laminin-related proteins, were originally identified as axonal guidance molecules. Subsequently, netrins were found to modulate various biological processes including morphogenesis, tumorogenesis, adhesion, and, recently, angiogenesis. In human placenta, the most vascularized organ, the presence of netrins has also been reported. Recent studies demonstrated the involvement of netrins in the regulation of placental angiogenesis. In this review we focused on the role of netrins in human placental angiogenesis. Among all netrins examined, netrin-4 and netrin-1 have been found to be either pro- or antiangiogenic factors. These opposite effects appear to be related to the endothelial cell phenotype studied and seem also to depend on the receptor type to which netrin binds, that is, the canonical receptor member of the DCC family, the members of the UNC5 family, or the noncanonical receptor members of the integrin family or DSCAM.

## 1. Vascular and Nervous Systems Similarities

The vascular and nervous systems share similarities at the anatomical and cellular levels: indeed both systems are a web of highly branched and complicated networks. The vessel system uses specialized tip cells which are located at the front of navigating blood vessels and which are morphologically and functionally similar to the axonal growth cone [1]. Moreover, both the vascular and nervous systems appear early during the embryonic development and evolve throughout the entire life. These observations have promoted investigations looking for the presence of guidance molecules outside the nervous cells; this led to the discovery of some axon guidance factors. Among these factors are netrins, which have since been shown to play a key role in the angiogenesis processes [2–4].

## 2. Netrins (Figure 1)

Netrins belong to a family of laminin-related proteins. All netrins comprise an N-terminal laminin-type domain which is followed by several epidermal growth factor-like (EGF) domains and a positively charged C-terminal domain. Five members of the netrin family have been identified in vertebrates. Two of them (netrin-G1 and netrin-G2) are bound to the cell membrane via glycosylphosphatidylinositol anchors [5]. Three are secreted proteins, including two netrins structurally related to the *γ* chain of laminin (netrin-1 and netrin-3) [6, 7] and one related to the laminin *β* chain (*β* netrin also known as netrin-4) [8, 9]. Netrins were initially described as regulators of axonal guidance during embryogenesis and are so called because netrin means one who guides in Sanskrit [6].

Besides the central nervous system, netrin expression was also reported in pancreas, lung, breast, and recently in the placenta [10, 11]. Netrins have been shown to play various important roles in central biological processes including cell guidance, adhesion, differentiation, and survival and recently in angiogenesis. Netrin-1 and netrin-4 are the most extensively studied members of the netrin family [12].

Netrin-1 is closely related to the laminin chain [13, 14]. The human netrin-1 gene is localized at 17p13-p12 [15] and encodes 604 amino acid protein of 70–84 kDa. It is a secreted protein that is involved in axonal outgrowth and migration orientation during the development of the central nervous system [14]. Netrin-1 was also reported to control morphogenesis of endothelial cells and vascular smooth-muscle cells. Bouvrée et al. demonstrated that netrin-1 inhibits sprouting angiogenesis in cloned chick through UNC5B-binding [16]. Netrins are also involved in cytoskeleton reorganization and in epithelial cell adhesion and migration in lung, mammary gland, and pancreas [4, 17–20], as well as in tumor growth [21] and in the regulation of inflammation [22].

The human netrin-4 gene is localized in 12q22-q23 [8] and encodes a 629-amino acid long protein of approximately 70–84 kDa. Netrin-4 is a secreted protein involved in neurite growth [8] and migration orientation during the development of the central nervous system [12, 23]. Besides the central nervous system, netrin-4 has been shown to regulate epithelial branching and morphogenesis in the lung [24], pancreas [25], salivary gland branching [26], lymphangiogenesis, angiogenesis, and tumor growth [27]. It was recently shown that netrin-4 promotes mural cell adhesion and recruitment to endothelial cells [28].

## 3. Netrin's Receptors

As far as we know, the biological effects of netrin-1 and netrin-4 are mediated through two different classes of transmembrane receptors. The DCC (deleted in colorectal cancer) family includes the DCC and neogenin receptors. These receptors mediate the attraction response of axons to netrins. The second family is the UNC5 (uncoordinated-5 homolog) family receptors which are responsible for mediating axonal repulsion (as a response to UNC5B homodimer) or axonal attraction (as a response to UNC5B and DCC heterodimers) [13, 29, 30]. In addition, in lymphatic endothelial cells, netrin-4 was shown to bind with high affinity to two different noncanonical receptors, namely, *α*6*β*1 integrin and laminin-1, both inducing local adhesion [23]. Whereas netrin-4 stimulates endothelial cell adhesion and migration, no effects were observed when UNC5B or neogenin was inactivated by siRNA [23]. These findings indicate that the initial step of netrin signaling pathway is more complex than the model of a single ligand signaling through a single receptor binding. Larivée et al. showed that UNC5B activation inhibits sprouting angiogenesis, and this data indicate that UNC5B is a potential antiangiogenic target [31].

Finally, disruption of one netrin-1 allele or of its UNC5B receptors was reported to be lethal during early embryogenesis in mice, suggesting a crucial role for netrin-1 and its UNC5B receptors in this process [32, 33].

## 4. Netrins and Angiogenesis

### 4.1. Netrin-1

In 2004, Anne Eichmann and colleagues reported the role of netrin-1 and its receptor UNC5B in controlling morphogenesis of the vascular system [4]. As a matter of fact, upon addition of netrin-1 to aortic ring in* in vitro* cultures, these authors observed inhibition of filopodia formation and sprouting, suggesting that netrin-1 is an antiangiogenic factor [1, 16, 31]. However, pro- and antiangiogenic activities were reported later on to be cell type dependent, presumably because of the existence of different patterns of angiogenesis-controlling genes expression. In this regard, other studies brought evidences for proangiogenic effects of netrin-1 in human umbilical vein endothelial cells (HUVEC) and human umbilical and arterial endothelial cells (HUAVEC) by demonstrating an increase in the proliferation, migration, tubal formation, and capillary branching after exposure to netrin-1 [18]. In another report, netrin-1 was found to promote angiogenesis through the control of the endothelial cells survival due at least in part to the apoptosis blockade induced by its unbound UNC5B receptor [34].

### 4.2. Netrin-4

Another netrin family member, netrin-4, was first investigated by Plouet et al. in HUVEC and HUAVEC cells. These authors showed that netrin-4 gene is specifically overexpressed in VEGF-stimulated endothelial cells* in vitro *as well as* in vivo* [3]. Knockdown of netrin-4 expression in these endothelial cells increased their ability to form tubular structures on Matrigel [3]. In contrast to netrin-1, netrin-4 binds only to neogenin but not to UNC5B or UNC5C receptors. Neutralization of netrin-4 binding to neogenin using blocking antibodies abolished the chemotactic effect of netrin-4. Furthermore, the silencing of either neogenin or UNC5B abolished netrin-4 inhibitory effect on endothelial cell migration, suggesting that both receptors mediate this function* in vitro*. Finally, netrin-4 significantly reduced tube formation structure on Matrigel and laser-induced choroidal [3] These observations led the authors to conclude that netrin-4 acts as an antiangiogenic factor through binding to neogenin and recruitment of UNC5B. Other studies have provided further support to this conclusion. As a matter of fact, Nacht et al. demonstrated that netrin-4 markedly inhibits endothelial cells migration and tube formation [35]. Moreover netrin-4 was found to have only negligible effects on endothelial cell proliferation [35]. In contrast, Mehlen's group showed that netrin-4 significantly protected HUVECs and HUAECs from serum deprived-induced apoptosis, as measured by the caspase-3 activity assay [2]. Endothelial cell migration was also studied and revealed that netrin-4 stimulated HUVECs migration. Furthermore, netrin-4 significantly induced angiogenesis in a dose-dependent manner with the optimal effect being observed at 150 ng/mL of netrin-4. These results demonstrate that netrin-4 can promote endothelial cell survival, proliferation, migration, and angiogenesis using* in vitro* systems [2].

**Figure 2:**
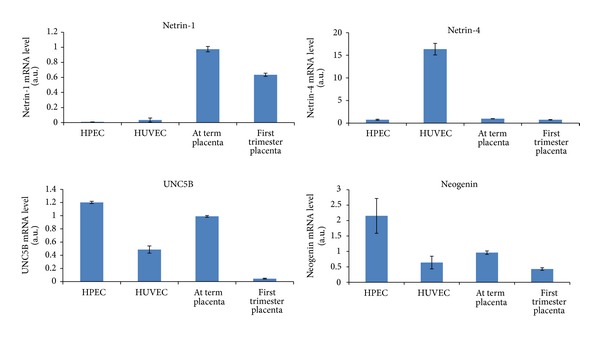
Netrin-1, netrin-4, UNC5B, and neogenin mRNA expression in first trimester and at term placenta and in endothelial cells HPEC and HUVEC normalized to term placenta (data not published).

From this short literature review, no clear consensus arises concerning the precise roles played by netrin-1 and netrin-4 in placental angiogenesis. Some studies described these signals as promoters of angiogenesis, whereas others stated opposite conclusions. These discrepancies in the available data leave open the question on the potential role of netrins in the control of angiogenesis [4, 36]. A possible explanation for these discrepancies could be the heterogeneity of the endothelial cell population studied [37]. Most of the investigations on the role of netrins in angiogenesis were performed using HUVEC; these cells have been used as a model for endothelial cells in many studies that considered placental angiogenesis. However, nowadays, growing literature shows that the placental macrovascular endothelial cells differ in phenotype, gene expression, and physiology from the microvascular endothelial cells, such as those present within the placental villi (HPEC) [37]; another possible explanation for the above-mentioned discrepancies is the receptor type to which netrins bind in the placenta, which is still not clear. As all receptors seem to be present in the placenta, further analyses are needed to determine the netrin receptors that are involved in the angiogenic processes. A last explanation could be a variation in specific mRNA levels between endothelial cells of different lineage; in fact, in our laboratory we were astonished to note a difference in mRNA expression of netrin-1, netrin-4, UNC5B, and neogenin between HPEC and HUVEC cells (Figure 2). Other investigators reported variation in mRNA level between HUVEC harvested in their laboratory and HUVEC purchased from American Type Collection [38]; in fact ATCC proposes both HUVEC (CRL-1730) and primary umbilical vein endothelial cells, which have different characteristics.

## 5. Therapeutic Potential of Netrins

A study of Delloye-Bourgeois et al. in 2009 has shown that netrin-1 inactivation induced vessel loss and inhibited primary tumor growth and metastases in animal models [39, 40]. Recently, the same team found that combining conventional chemotherapies with netrin-1 interference could be a promising therapeutic approach [41]. On the other hand, netrin-4 overexpression decreased tumor recurrence and metastases after surgical resection in mouse models [40, 42, 43]. All these data suggest that inactivation of netrin-1 and overexpression of netrin-4 can be useful in tumor therapy. Other studies indicated that netrin-1 inhibits migration of monocytes, neutrophils, and lymphocytes via its receptors UNC5B. Another study by Zhang and Cai in 2010 demonstrates that netrin-1 potently protects the heart from I/R injury by stimulating NO production from cardiac ECs and myocytes [44]. Concerning the relationship between inflammation and netrin-1 van Gils et al. in 2010 established that netrin-1 inhibited macrophage migration via UNC5B, in case that the presence of an atherosclerotic plaque and that deletion of netrin-1 in myeloid cells severely reduced atherosclerosis lesion size [45, 46].

Contrary to these studies, others reported that netrin-1 reduced ischemia-reperfusion injury by decreasing apoptosis in endothelial cells and that netrin-1 enhanced focal neovascularization, reduced infarct size, and improved long-term functional recovery after transient focal cerebral ischemia [47]. Hence, netrin-1 can serve as an innovative agent for the treatment of strokes. Durrani et al. have shown that, by combining an increase of angiogenesis and a decrease of cardiomyocytes apoptosis, netrin-1 effectively reduced ischemia-reperfusion injury to preserve global heart function [48].

All these studies demonstrated dual roles for netrin-1. The question remains whether netrin-1 therapies using netrin-1 treatment or inactivation of netrin-1 give clinical benefit compared to acute side effects such as loss or unwanted angiogenesis or apoptosis.

## 6. Netrins in the Placenta (Figure 3)

We recently investigated and characterized the expression of netrin-1 and its receptors DCC and UNC5B in the human placenta [10]. Coexpression of both netrin-1 and UNC5B in villous cytotrophoblasts and endothelial cells suggested an autocrine regulatory mechanism in these cells [10]. We have also demonstrated that netrin-1 plays a key role in cytotrophoblast proliferation and found that gene expression of UNC5B is upregulated by hypoxia [49] Ramkhelawon et al. also showed an upregulation of UNC5B by hypoxia in macrophage and also an upregulation of netrin-1 by hypoxia via HIF [50].

In a recent investigation, we demonstrated the expression and the cellular localization of netrin-4 and neogenin in human first trimester and term placenta [11]. Netrin-4 was found in the syncytiotrophoblast, together with neogenin. On the other hand, villous cytotrophoblast cells, which we had previously described as expressing the UNC5B receptor [10], also express netrin-4 [11]. Moreover, we observed that villous cytotrophoblast cells did not express neogenin. Other localizations of netrin-4 were observed in proximal extravillous and distal invasive cytotrophoblast cells. However, because neogenin staining was absent from these cells, it became clear that netrin-4 effects, if any, are mediated through receptors other than neogenin. Neogenin was however expressed by villous mesenchymal cells. We also showed a strong netrin-4 and neogenin expression in placental endothelial cells suggesting that netrin-4 might have both paracrine and autocrine signals in these cells [11].

## 7. Placental Angiogenesis

During placental development, angiogenesis follows vasculogenesis and leads to the remodeling of the vascular plexus into a branched vascular tree to ensure increased nutritional and gas exchanges and efficient elimination of fetal waste. This placental angiogenesis is dependent on various growth factors including the vascular endothelial growth factor (VEGF), the placental growth factor (PlGF), and the basic fibroblastic growth factor (bFGF) [51]. More recently, the axonal guidance molecules, netrins, have also been suggested to play a key role in the regulation of angiogenic processes. Importantly, the most threatening placental pathology, the preeclampsia, has been reported to be associated with alterations in the expression of these factors [52]. The present review focuses on the potential roles of netrins and their receptors in placental angiogenesis.

## 8. Netrins and Placental Angiogenesis

It was also observed that netrin-1 accelerated neovascularisation in the placenta of gravid rats [53]. More importantly, suppression of netrin-1 expression in the placenta resulted in reduced vascular sprouting* in vivo*. These findings suggest that netrin-1 is essential for the proper functioning of HUVECs and for angiogenesis in rat placenta and appears therefore to be necessary for placental and foetus development [53, 54]. Exposure of human placental microvascular endothelial cells (HPECs) to netrin-1 also resulted in enhanced cell viability, migration, and tube formation [54]. Taken together, these observations provide strong support for a key role of netrin-1 as a promoter of blood vessels formation in human placenta. This is an additional argument for netrin-1 to be a potential target for new therapeutic strategies in placental vasculature-related diseases.

Further lines of evidence for a proangiogenic effect of netrin-1 in HUVECs were reported by Xie et al. [53]. Involvement of netrin-1 in placental pathologies was first evoked by Yang et al. who reported decreased netrin-1 mRNA and protein expressions together with a reduced vascular density in term placenta from pregnant women with preeclampsia [52]. Moreover, Qian-Hua et al. demonstrated that netrin-1 expression was significantly reduced in placenta from women bearing foetuses with growth restriction when compared to pregnant control women [55]. Furthermore, as netrin-1 was shown to enhance viability of HPECs and to inhibit their apoptosis, it can be proposed that this protein controls vascular growth in the placenta and that failure in its expression might be associated with the development of placental pathologies such as fetal growth restriction [55].

## 9. Conclusions

In summary, netrins are present in the placenta and are important for placental development (proliferation of cytotrophoblast) and also for placental vascular development, but what about their role in maternal vascular adaptation to pregnancy?

Here, we highlighted the roles of netrins as angiogenic factors in the placenta. However, the molecular mechanisms underlying netrin effects remain largely unknown in human placenta. Therefore, additional research may provide new insights into the role of netrins in normal human placenta and associated diseases, such as preeclampsia and growth restriction, which are both characterized by failures in angiogenesis processes.

## Figures and Tables

**Figure 1:**
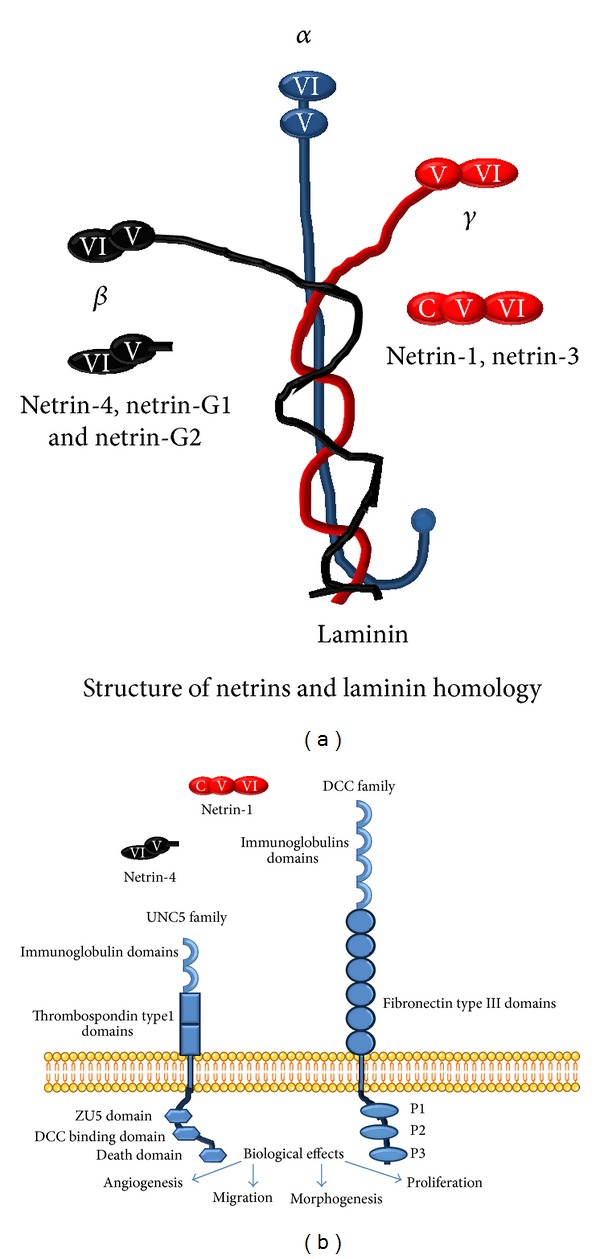
Netrins and their receptors. (a) Netrins show homology with laminin. Netrin-1 and netrin-3 are related to the *γ* chain of laminin, and *β* netrin, also known as netrin-4, is related to the laminin *β* chain. (b) Netrins bind to receptor members of the DCC family and to members of the UNC5 family. Their most important roles are given [12].

**Figure 3:**
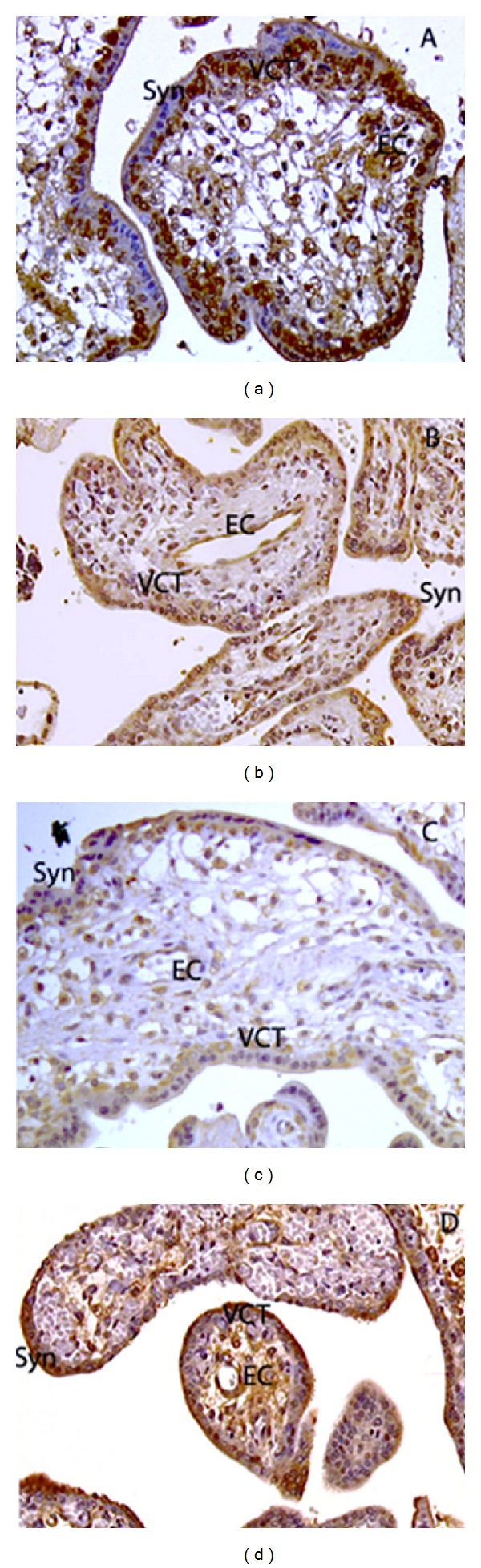
Immunohistochemical localization of netrin-1 (a), netrin-4 (b), UNC5B (c), and neogenin (d) in human first trimester placenta. EC: endothelial cells; Syn: syncytiotrophoblast; VCT: villous cytotrophoblasts.
